# Assessment of Motor Activities of Daily Living: Spanish Cross-Cultural Adaptation, Reliability and Construct Validity of the DCDDaily-Q

**DOI:** 10.3390/ijerph17134802

**Published:** 2020-07-03

**Authors:** Laura Delgado-Lobete, Rebeca Montes-Montes, Berdien W. van der Linde, Marina M. Schoemaker

**Affiliations:** 1 University of A Coruña, Faculty of Health Sciences, Health Integration and Promotion Research Unit (INTEGRA SAÚDE), 15011 A Coruña, Spain; l.delgado@udc.es; 2 University of Groningen, University Medical Center Groningen, Centre for Human Movement Sciences, 9713 GZ Groningen, The Netherlands; m.m.schoemaker@umcg.nl; 3 University of A Coruña, TALIONIS Research Group, Research Centre of the Galician University System, Centre for Information and Communications Technology Research (CITIC), 15008 A Coruña, Spain; 4 Hanze University of Applied Sciences, Centre of Expertise Healthy Ageing, 9747 AS Groningen, The Netherlands; b.w.moraal@pl.hanze.nl

**Keywords:** developmental coordination disorder, DCDDaily-Q, cross-cultural adaptation, validity, reliability, screening, parental questionnaire

## Abstract

The DCDDaily-Q is an instrument that aims to comprehensively assess motor performance in a broad range of activities of daily living (ADL) and to identify risk of Developmental Coordination Disorder (DCD) in children. The aim of this study was to cross-culturally adapt the DCDDaily-Q into European Spanish (DCDDaily-Q-ES) and to test its psychometric properties in Spanish 5 to 10 year old children. The DCDDaily-Q was translated and cross-culturally adapted into Spanish following international guidelines. Two-hundred and seventy-six parents of typically developing Spanish children completed the final version of the DCDDaily-Q-ES (M = 7.5 years, SD = 1.7; girls = 50%). Confirmatory Factor Analysis (CFA), internal consistency, and corrected item-total correlations were conducted to test construct validity, internal consistency, and homogeneity of the DCDDaily-Q-ES. The DCDDaily-Q-ES achieved good semantic, conceptual, and cultural equivalence. CFA supported construct validity of the DCDDaily-Q-ES. Reliability values were also good (Cronbach’s alpha = 0.703–0.843; corrected item-total correlations = 0.262–0.567). This is the first study to cross-culturally adapt and examine the DCDDaily-Q outside the Netherlands. The findings suggest that the DCDDaily-Q-ES is a reliable and valid measure to assess learning, participation, and performance in a broad range of ADL.

## 1. Introduction

Developmental Coordination Disorder (DCD) is one of the most prevalent neurodevelopmental disorders in children, as it is present in 5–6% of schoolchildren [1,2]. According to the Diagnostic and Statistical Manual of Mental Disorders, 5th edition (DSM-5), a child should be diagnosed with DCD if (criterion A) the acquisition and performance of their coordination motor skills are substantially below that expected for their chronological age and opportunity for skill learning and use; (criterion B) this motor skills deficit significantly and persistently interferes with participation in activities of daily living (ADL), including self-care, school productivity or fine motor activities, and leisure or gross motor play activities; (criterion C) the motor skills deficit occurs in the early developmental period; (criterion D) the motor skills deficit cannot be better explained by a neurological or developmental condition affecting movement [1,2].

Early identification of DCD is highly recommended, as this may prevent further health consequences and promote intervention as soon as the disorder is detected, adding to its prevention [2,3,4]. There are several screening tests to operationalize criterion B, with the Developmental Coordination Disorder Questionnaire (DCDQ) being the most frequently used and recommended questionnaire [2,5]. However, the DCDQ does not evaluate participation or performance in specific self-care ADL. Assessing self-care and self-maintenance ADL when evaluating DCD is needed as this disorder has a great impact on self-care functioning [6,7,8,9,10].

Recent research suggests that the prevalence of DCD in Spanish children is high, as 8–12% of schoolchildren show risk of DCD [11,12,13]. Until now, the DCDQ is the only cross-culturally adapted and valid questionnaire available to identify children with probable DCD in Spain [14,15], but a measure that assesses both participation and quality of motor performance in a broad range of meaningful, functional daily activities in Spain is lacking. Additionally, academic researchers and health practitioners could benefit from having an instrument that addresses both performance and participation to further promote performance through participation in children with motor coordination issues, as recommended by the International Classification of Functioning, Disability, and Health [16].

The DCDDaily-Questionnaire (DCDDaily-Q) is the first parent questionnaire that extensively covers the broad range of ADL that children with DCD struggle with in their daily life [17]. This measure does not only assess quality of motor performance but also whether children actually participate in the ADL, and how long it took them to learn each activity. The original version of the DCDDaily-Q is aimed at children aged 5–8 years. It shows excellent psychometric properties and discriminant capacity, and several studies have highlighted its potential in screening children at risk of DCD [2,17,18]. Additionally, the DCDDaily-Q shows good criterion validity with both the Dutch and the Spanish versions of the DCDQ (r = 0.638 and r = 0.406, respectively) [17,19].

However, no study has provided cross-cultural adaptation and validation of this measure outside the Netherlands or tested whether it could be used for children older than eight years. Prior to using the DCDDaily-Q in another cultural context, it is necessary to test its cultural equivalence and psychometric properties in a representative sample of the target population [20,21]. Therefore, the purpose of this study was to cross-culturally adapt and examine the reliability and construct validity of the DCDDaily-Q in Spanish 5 to 10 year old children.

## 2. Materials and Methods

**Figure 1:**
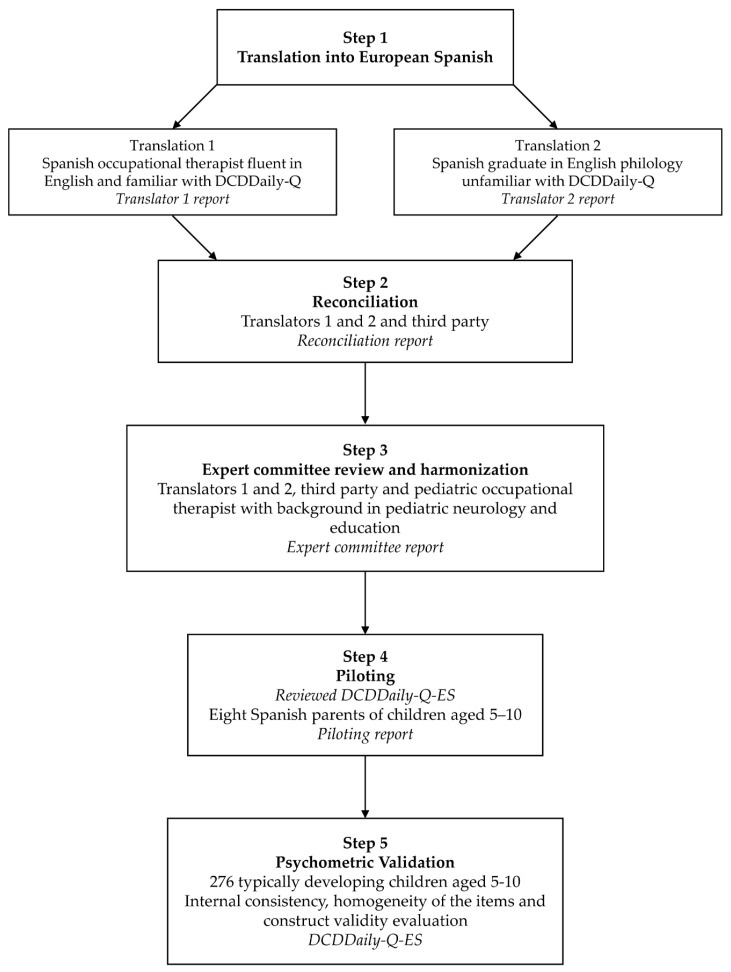
Cross-cultural and psychometric validation process of the Spanish DCDDaily-Q.

We conducted a cross-cultural adaptation and validation study of the DCDDaily-Q in a Spanish population following international guidelines [20,21,22,23,24] (Figure 1).

### 2.1. Cross-Cultural Adaptation

Consent to cross-culturally adapt and validate the DCDDaily-Q to European Spanish was received from the original developers, while ethical approval was obtained from the Autonomic Research Ethics Committee of Galicia in December 2018 (code 2018-606). As there is not a consensus for cross-cultural adaptation guidelines, we followed the most recommended and commonly used guidelines, and included the most significant steps, without which, the cross-cultural adaptation could not be reliable—two independent forward translations, synthesis, expert committee review, and piloting in target population [20,22,23,24].

#### 2.1.1. Step 1. Two Independent Forward Translations

Two independent forward translations into European Spanish were made, one conducted by an occupational therapist fluent in English and familiar with the DCDDaily-Q and DCD (translator 1 = T1), and the other one performed by a professional translator unfamiliar with the DCDDaily-Q or DCD (translator 2 = T2). Each translator was asked to rank the difficulty they found when trying to translate each item into Spanish, while keeping its original meaning from 0 to 10 (low difficulty, 0–3; moderate difficulty, 4–6; high difficulty, 7–10).

#### 2.1.2. Step 2. Reconciliation of the Forward Translations

Reconciliation between the two initial forward translations into one single translation was performed using a third-party approach [24]. Both initial translators and an occupational therapist with a background in cross-cultural adaptation research and familiar with the DCDDaily-Q compared the independent forward translations and discussed all the questionnaire sections (i.e., instructions, final comments) and items to elaborate a single forward translation.

As suggested by the International Society for Quality of Life Research (ISOQOL) Translation and Cultural Adaptation Special Interest Group, decision criteria for the single translation were guided by source and comprehensibility, cultural, grammatical, and terminology reasons [24].

#### 2.1.3. Step 3. Expert Committee Review and Harmonization

The expert committee comprised the two initial translators, the third party involved in the reconciliation step, and one occupational therapist proficient in pediatric neurological rehabilitation, who was also a special education teacher. All participants were asked to suggest changes that they considered could improve the translation quality. Once their suggestions had been implemented, they were asked to rank the final items equivalence as A (both semantically and conceptually equivalent), B (conceptually equivalent, though minor semantic changes are present) or C (the item could not be considered equivalent).

Additionally, in order to address the potential utility of the DCDDaily-Q in children older than 8 years, the three occupational therapists involved in the expert committee review and harmonization (i.e., T1, E3 and E4) addressed each one of the activities of the questionnaire to determine whether they were relevant and significant for older children. The three experts concluded that the activities were appropriate to evaluate the everyday performance of children aged 9–10 years, but not of children older than 10 years. Therefore, the piloting and psychometric validation of the Spanish version of the DCDDaily-Q included children aged 5–10 years.

#### 2.1.4. Step 4. Piloting/Cognitive Debriefing Interviews

The reviewed questionnaire was trialed on a community-based convenience sample of eight mothers of children aged 5 to 10 from different regions and educational and occupational backgrounds, using one-to-one cognitive debriefing interviews [21]. Piloting is a necessary step in the cross-cultural adaptation of any measure as it aims to check for misunderstandings, incomplete concept coverage and inconsistent interpretations [22].

### 2.2. Psychometric Validation

Internal consistency, homogeneity of the items, and construct validity were evaluated using a stratified random selected sample of 276 typically developing children aged 5 to 10, without known developmental disorders, that attended seventeen different schools in three Spanish regions (M = 7.5 years, SD = 1.7; girls = 50%; northwest = 52.9%, north = 42.7%, central = 4.4%) (Table 1). A sample size of at least 200 participants was needed in order to conduct a confirmatory factor analysis (CFA) achieving minimal bias (< 0.05) and a statistical power > 0.8, considering that the DCDDaily-Q has a three-factor structure with at least six items per factor [25]. The DCDDaily-Q-ES was sent to the parents of the participants between December 2018 and December 2019 via school intermediation. Parents anonymously and voluntarily returned it to the schools after completion within one week. The first two authors then retrieved the questionnaires from the schools.

### 2.3. DCDDaily-Questionnaire

The DCDQDaily-Q is a 23-item parent questionnaire, of which the main purpose is to assess motor performance of daily living activities in children aged 5–8 years, but this instrument also measures the grade of participation of the child in the ADL and how long it took them to learn each activity [16]. The DCDDaily-Q was originally developed in the Netherlands and it is available in both Dutch and English. Its validation study reported excellent psychometric properties and discriminant validity (Cronbach’s alpha = 0.85; sensitivity = 88%; specificity = 92%) [17].

The DCDDaily-Q comprises 23 items referring to three subscales that assess different daily living activities—self-care activities (items 1 to 10; e.g., item 1 = buttering a sandwich), fine motor activities (items 11 to 17; e.g., item 11 = writing) and gross motor activities (items 18 to 23; e.g., item 18 = playing hopscotch). The items included in the questionnaire were designed in order to address the daily activities that children with DCD struggle more often with [17].

Motor performance is assessed with a 3-point Likert scale ranging from 1 (good performance) to 3 (poor performance), while participation is measured with a 4-point Likert scale ranging from 1 (the child does the activity regularly) to 4 (the child never does the activity). Higher scores indicate poorer performance or less participation, respectively. Additionally, parents are asked to indicate if the child took longer to learn the activity than their peers in a yes/no response, where 1 = yes and 0 = no.

### 2.4. Data Analysis

The IBM Statistical Package for Social Sciences version 25 (SPSS Inc., Chicago, IL, USA) and EQS-Structural Equation Modeling Software version 6.1. were used for the analysis. Internal consistency of the DCDDaily-Q-ES was assessed using Cronbach’s alpha coefficient with values above 0.70 and 0.80 being considered adequate and good, respectively. Corrected item-total correlations were performed to test homogeneity of the items, with values above 0.30 being considered adequate. Construct validity for the original three-factor structure of the DCDDaily-Q was tested using a robust unweighted least squares estimation method [26,27,28,29]. A Satorra chi-square (SX^2^)/degrees of freedom ratio value less than five, a Root Mean Square Error of Approximation (RMSEA) value of <0.08, and Comparative Fit Index (CFI) and Non-Normed Fit Index (NNFI) values of >0.90 indicated an adequate fit of the model [30,31,32].

## 3. Results

### 3.1. Cross-Cultural Adaptation

Both translators found the items of DCDDaily-Q easy to translate (T1 = 1.9, SD = 0.7; T2 = 0.6, SD = 0.7) (Table 2). Only item 18 (playing hopscotch) was ranked as moderately difficult to translate by one translator. Regarding reconciliation and harmonization of items, Appendix A Table A1 shows an example of two items’ reconciliation and expert review/harmonization using the approach proposed by the ISOQOL Translation and Cultural Adaptation Special Interest Group.

After these steps, item syntax underwent semantic changes to make the reading sound more natural in Spanish (e.g., starting the item with “to use controlled motions” instead of “controlled motions”), to add supplementary explanations or examples (e.g., specifying “pouring juice/water/milk” instead of “pouring juice”) or to adapt them to the Spanish cultural characteristics (e.g., using “buttering toast” instead of “buttering a sandwich”). Every item was considered both semantically and conceptually equivalent by at least one expert after review and harmonization, so no further modifications were needed. Additionally, there was 69.57% A rated agreement between all four experts (the item is both semantically and conceptually equivalent) and no item was ranked as C (the item could not be considered equivalent) (Table 2).

All participants in the piloting trial considered the items well written and that instructions were clear. None of them expressed any difficulty or misunderstanding or considered that any section needed further modifications or additional examples. Finally, all the activities included in the questionnaire were familiar to the participants. Therefore, the final step of the cross-cultural adaptation did not lead to further modifications of the final version of the DCDDaily-Q-ES.

An example of the scoring of the DCDDaily-Q-ES items is provided in Appendix A (Table A2).

### 3.2. Psychometric Properties

Overall, DCDDaily-Q-ES internal consistency was good (Cronbach’s alpha = 0.843); subscales reported adequate Cronbach’s alpha values as well (self-care ADL = 0.703; fine motor ADL = 0.725; gross motor ADL = 0.703), indicating good internal consistency. Most of the items showed adequate corrected item-total correlations. Only items 5, 6, 16 and 17 scored below the recommended cutoff criteria, but were close to 0.30 (0.262–0.298). Cronbach’s alpha did not increase if any of the DCDDaily-Q-ES items were deleted, therefore, suggesting that no item was problematic (Table 2).

**Figure 2:**
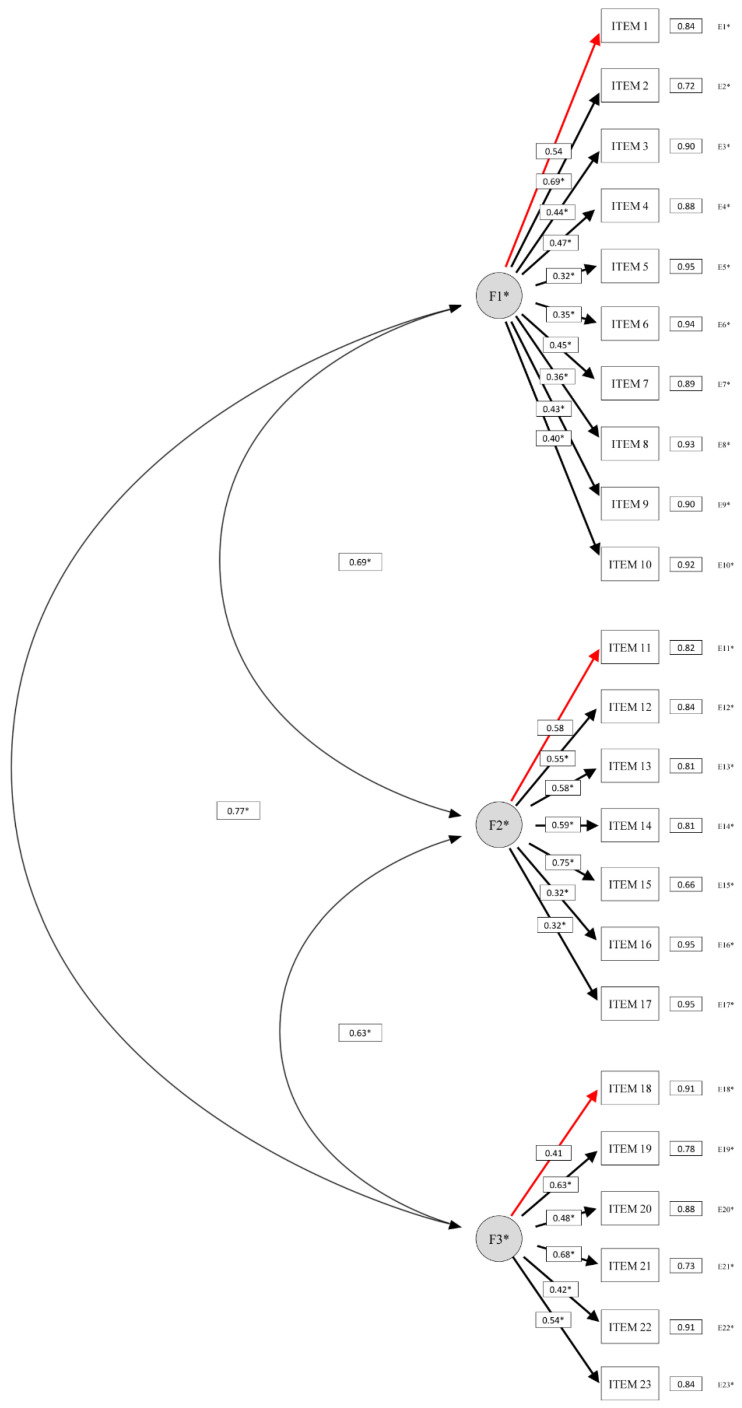
Confirmatory factor analysis of the Spanish DCDDaily-Q three-factor model with standardized estimates (*n* = 276). Items 1, 11 and 18 fixed to 1 during estimation. * = *p* < 0.05; double-headed curved arrows—correlations between factors; F1—self-care; F2—fine motor; F3—gross motor; E—error.

Regarding the construct validity of the Spanish DCDDaily-Q, the original three-factor model demonstrated a good fit to the data (SX^2^ (227) = 405.86, *p* < 0.05; SX^2^/df = 1.79; CFI = 0.940; NNFI = 0.933; RMSEA = 0.054, 90% CI = 0.045–0.062). All the loadings were significant and ranged from 0.32 to 0.75 (Figure 2). Moreover, the correlation values between the hypothesized latent factors demonstrate a significant, moderate to strong correlation between self-care, fine motor, and gross motor activities (0.63–0.77).

## 4. Discussion

There is very limited research about motor coordination problems or DCD in Spanish children, partially due to the lack of reliable, valid and easily accessible instruments to measure motor performance in daily living. The present study describes the cross-cultural adaptation and psychometric validation of a parent questionnaire aimed to assess performance and participation of a broad range of motor-based, functional, everyday activities.

### 4.1. Cross-Cultural Adaptation

During both reconciliation and expert committee review of the DCDDaily-Q-ES, several semantic and conceptual issues were addressed, leading to a more compelling and culturally adapted final questionnaire than any of the first two forward translations. Cross-cultural and language differences between countries make it necessary to follow rigorous translation, equivalence reviewing, and piloting in the target population when adapting a questionnaire to a new context [20,23]. While there is no consensus about the cross-cultural adaptation process, research has shown that two or more independent forward translations, reconciliation, expert committee review, and cognitive debriefing interviews should be included in the process [22,23].

Although both translators found low difficulties when translating the DCDDaily-Q-ES, the translator familiar with both the DCD and the DCDDaily-Q consistently considered it to be more difficult than the translator unfamiliar with both the DCD and the DCDDaily-Q. When discussing it during the reconciliation step, T1 stated that as she was familiar with the topic and the measure, she sometimes struggled more to find the term or rephrasing that better reflected the original intended meaning in terms of movement aspects, while T2, who was a professional translator, was more concerned about making the most technically adjusted translation. Overall, the third-party implication and the opportunity given to T1 and T2 to discuss these aspects improved the reconciled translation, which is in line with the recommendations of the ISOQOL Translation and Cultural Adaptation Special Interest Group [24]. The use of both familiar and unfamiliar translators has been useful to significantly improve the equivalence in other DCD related questionnaires in the Spanish population [14].

Recording difficulty in translation between translators is not a common practice in cross-cultural adaptation studies of DCD-related instruments, but in this case, it suggests that familiar translators may adopt a more conservative approach, adding to the evidence of the necessity of performing, at least, two independent translations with different translator profiles, and then, reconcile or synthesize them [22,23,24].

Overall, both the expert committee and the parents included in the piloting reported an excellent cultural adaptation of the Spanish version of the DCDDaily-Q. While grammatical changes were necessary, very few cultural issues were found during the forward translations and expert committee review, as only minor changes were undertaken to make the activities look more familiar to Spanish children without losing its intended meaning (i.e., items 1 and 3). This could be explained by several factors. In the first place, both the original and target population are in Western, European countries that are expected to share common experiences, especially regarding self-care and productive daily activities. Additionally, this could point towards a cultural invariance of the DCDDaily-Q, suggesting that the daily activities included in the questionnaire are relevant and significant for children from different countries and cultural backgrounds.

### 4.2. Psychometric Properties

The results concerning reliability (i.e., internal consistency and homogeneity of the items) and construct validity showed that the Spanish version of the DCDDaily-Q is a reliable and robust questionnaire. The DCDDaily-Q-ES demonstrates a good internal consistency, with a Cronbach’s alpha of 0.843 for the total scale and above 0.70 for the three subscales. This outcome is consistent with the internal consistency value reported in the psychometric validation study of the DCDDaily-Q (Cronbach’s alpha = 0.85) [17].

Four items showed corrected item-total correlations below 0.30, which could suggest poor homogeneity of those items (i.e., items 5, 6, 16 and 17). However, the corrected item-total correlations were above 0.20 and close to 0.30 [33,34]. Additionally, the activities addressed in those four items are very common ADL in Spanish schoolchildren (item 5 = eating soup; item 6 = washing hands; item 16 = Lego building; and item 17 = moving pawns) and their removal did not lead to the improvement of Cronbach’s alpha, so the decision was to keep them in the final version of the questionnaire.

Confirmatory factor analysis supported the robustness of the original three-factor structure, reflecting the broad and diverse range of applicable ADL included in the DCDDaily-Q [17]. This is particularly relevant because the DCDDaily-Q is meant to be answered by parents, so the activities included in the questionnaire need to cover different occupational areas which are significant to the child (i.e., motor-based school activities), but which can be observed by parents (i.e., writing, coloring, using scissors or folding paper sheets). The moderate to strong correlations found between the hypothesized latent factors (i.e., self-care, fine motor, and gross motor activities) further support the findings from the Exploratory Factor Analysis conducted in the original version [17]. Additionally, a significant and moderate correlation between the Spanish cross-culturally adapted versions of the DCDDaily-Q and the DCDQ has been reported in a previous study, providing evidence of its criterion validity (*p* < 0.001) [19].

Potential differences between performance and participation scores of the DCDDaily-Q across age have been previously addressed both in Dutch and Spanish children [17,19]. Significant differences between age groups were found in both populations, where parents of younger children reported poorer motor performance and lower daily participation. Therefore, specific cutoff points for age have been provided for Dutch and Spanish children [17,19].

To the best of our knowledge, this is the first study to cross-culturally adapt and validate the DCDDaily-Q in a new population outside the Netherlands. Our results provide further evidence of the reliability and validity of this instrument to assess motor-based daily activities in children. However, further studies are needed to adapt and validate the DCDDaily-Q in other populations. Instruments aimed to evaluate daily motor performance in order to assess for risk of DCD in children must be valid, reliable, and culturally adjusted to the target population. The DCDDaily-Q was developed to include those daily activities that children with DCD struggle with more often and significantly [17]. Therefore, the DCDaily-Q-ES is an easily accessible, reliable, robust and culturally adapted measure that can be recommended to assess motor performance and participation of ADL in Spanish children.

### 4.3. Study Strengths and Limitations

The main strength of this study is the rigorous and methodic process that was carried out in order to ensure the cross-cultural equivalence of the DCDDaily-Q in Spanish children, which also introduced novel aspects regarding the relevance and implications of using two independent translators in cross-cultural adaptation studies. The stratified random sampling method allowed for a more representative, sex and age-balanced sample. Additionally, this is the first study to confirm the psychometric properties of the DCDDaily-Q in children older than eight years, proving that this instrument can be used in children up to 10 years old.

This study has several limitations. The geographical distribution of the participants was not proportionate to the overall Spanish population, which may introduce potential bias. Correlation between the DCDDaily-Q-ES and an objective motor test, such as the Movement Assessment Battery for Children-2, was not explored. However, the concurrent validity between the DCDDaily-Q-ES and the Spanish version of the DCDQ has been demonstrated in a previous work [19]. Performance and participation of the children are parent-reported, which may introduce potential bias. On the other hand, previous studies have argued that parents are able to provide reliable and valuable information about their child’s everyday performance and participation, which may be difficult to determine in a clinical evaluation [17]. Another limitation is the lack of a sample of children clinically diagnosed with DCD, as this disorder is an extremely underdiagnosed condition in Spain [35]. Therefore, future studies are needed to assess the capacity of this instrument to discriminate clinical populations.

### 4.4. Implications for Clinical Practice and Future Research

Health practitioners working with children with motor coordination issues, like occupational therapists, could use the DCDDaily-Q to comprehensively assess the child’s difficulties in specific self-care, fine motor, and gross motor ADL, in order to set goals for the design of a task-oriented intervention aimed to promote participation. Having a similar instrument in different countries is useful for researchers as well, as it facilitates direct comparison of ADL participation and motor performance across countries. Furthermore, cross-cultural adaptation and validation of the DCDDaily-Q in new contexts would make it possible to carry on studies on DCD and motor coordination difficulties involving children from different countries.

## 5. Conclusions

The DCDDaily-Q-ES was cross-culturally adapted to the Spanish population and showed a good reliability and construct validity. These findings add to the evidence of the DCDDaily-Q as an appropriate measure for assessing a broad range of ADL learning, participation, and motor performance. This is the first study to explore the psychometric properties of this tool in children up to 10 years old. Future studies can expand on these findings by exploring the correlation between the DCDDaily-Q-ES and the MABC-2, and the discriminant capacity of the DCDDaily-Q-ES, including a clinical sample of children diagnosed with DCD. Overall, the DCDDaily-Q-ES is a free, user friendly questionnaire that can be utilized by researchers and health practitioners to address criteria B of the diagnostic criteria for DCD, and to identify functional, everyday challenges in children in order to design individual and specific treatment goals aimed to promote daily participation.

## Figures and Tables

**Table 1:** Characteristics of the psychometric validation participants (*n* = 276).

Participants	N	Girls (N (%))
5 years old	46	23 (50.0)
6 years old	46	23 (50.0)
7 years old	46	23 (50.0)
8 years old	46	23 (50.0)
9 years old	46	23 (50.0)
10 years old	46	23 (50.0)
Northwest	146	66 (45.2)
North	118	66 (55.9)
Central	12	6 (50.0)
Public school	146	73 (50.0)
Semi-private/private school	127	64 (50.4)

**Table 2:** Translation subjective difficulty, equivalence, and reliability properties of the DCDDaily-Q-ES.

DCDDaily-Q-ES	Translation Subjective Difficulty		Equivalence				Cronbach Alpha if Item Deleted	Corrected Item-TOTAL correlations	*Mean* (*SD*)
	T1	T2	T1	T2	E3	E4			
Item 1	1	1	B	B	B	A	0.836	0.429	1.5 (0.6)
Item 2	2	0	A	A	A	A	0.829	0.567	1.5 (0.7)
Item 3	2	1	B	B	B	A	0.838	0.400	1.2 (0.4)
Item 4	1	0	A	A	A	A	0.837	0.419	1.4 (0.5)
Item 5	2	1	A	A	A	A	0.842	0.273	1.1 (0.2)
Item 6	2	0	A	A	A	A	0.842	0.298	1.0 (0.2)
Item 7	2	0	A	A	A	A	0.838	0.391	1.3 (0.5)
Item 8	2	0	A	A	A	A	0.840	0.319	1.3 (0.5)
Item 9	3	0	A	A	A	A	0.840	0.362	1.6 (0.7)
Item 10	2	1	A	A	A	A	0.840	0.332	1.2 (0.4)
Item 11	2	0	B	A	A	B	0.835	0.460	1.3 (0.5)
Item 12	2	0	A	A	A	A	0.837	0.417	1.2 (0.4)
Item 13	1	1	A	A	A	A	0.835	0.444	1.3 (0.5)
Item 14	1	1	A	B	A	A	0.835	0.453	1.2 (0.5)
Item 15	1	0	A	A	A	A	0.831	0.567	1.4 (0.5)
Item 16	2	1	A	B	A	A	0.842	0.262	1.2 (0.4)
Item 17	3	2	B	B	B	A	0.842	0.267	1.1 (0.4)
Item 18	4	2	B	B	A	A	0.839	0.348	1.3 (0.5)
Item 19	1	1	A	A	A	A	0.832	0.514	1.7 (0.7)
Item 20	2	0	A	A	A	A	0.837	0.396	1.3 (0.5)
Item 21	2	0	A	A	A	A	0.831	0.563	1.4 (0.5)
Item 22	2	0	A	A	A	A	0.841	0.332	1.6 (0.6)
Item 23	2	1	A	A	A	A	0.837	0.442	2.0 (0.8)

T1—translator 1; T2—translator 2; E3—expert 3; E4—expert 4; A—the item is both semantically and conceptually equivalent; B—the item is conceptually equivalent though minor semantic changes are present.

## Appendix A

**Table A1:** Example of items reconciliation and revision.

**Item 3: Pouring juice. Controlled motions, without spilling or hitting the glass/table or dropping the cap, neatly opening and closing the bottle/container, at a normal pace.**	
T1	Servir un vaso de zumo/agua/leche. Emplea movimientos controlados a un ritmo normal, sin salpicar/derramar el líquido, golpear la mesa o dejar caer el vaso, y abre y cierra adecuadamente la botella o tetrabrik.
T2	Verter zumo en un recipiente. Movimientos controlados a un ritmo normal, sin derramar el líquido y sin que caiga el vaso o la taza, abriendo y cerrando cuidadosamente el envase.
Reconciled translation	Servir un vaso de zumo/agua/leche. Emplear movimientos controlados a un ritmo normal, sin salpicar/derramar el líquido, golpear la mesa o dejar caer el vaso, y abriendo y cerrando adecuadamente la botella o tetrabrik.
Decision criteria	Best reflects the conceptual definitions and meaning of the source text. Is understandable to a population of varied education levels. Reads more naturally in the target language.
Final translation after expert committee review	Servir un vaso de zumo/agua/leche. Emplear movimientos controlados a un ritmo normal, sin salpicar/derramar el líquido, golpear la mesa o el vaso ni dejar caer el tapón, y abriendo y cerrando adecuadamente la botella o tetrabrik.
Decision criteria	Best reflects the stress of the source text. Reads more naturally in the target language. Is semantically precise.
**Item 6. Washing hands. Tap is accurately opened and closed, hands cleaned well without spilling soap or water, and wiped dry, at a normal pace.**	
T1	Lavarse las manos. Abre y cierra el grifo de forma adecuada y se limpia y seca correctamente las manos, a un ritmo normal y sin salpicar agua ni dejar caer el jabón.
T2	Lavarse las manos. Abrir y cerrar el grifo correctamente, lavarse las manos sin que se caiga el jabón y sin salpicar a un ritmo normal, y secarse las manos.
Reconciled translation	Lavarse las manos. Abrir y cerrar el grifo de forma adecuada y limpiándose y secándose correctamente las manos, a un ritmo normal y sin salpicar agua o derramar el jabón.
Decision criteria	Best reflects the conceptual definitions and meaning of the source text. Reads more naturally in the target language. The syntax is correct.
Final translation after expert committee review	Lavarse las manos. Abrir y cerrar el grifo de forma adecuada, limpiarse y secarse correctamente las manos a un ritmo normal y sin salpicar agua ni derramar o que se le escurra el jabón.
Decision criteria	Best reflects the stress of the source text. Reads more naturally in the target language. The syntax is correct. Is semantically precise.

T1 = translator 1; T2 = translator 2.

**Table A2:** Illustrative item of the DCDDaily-Q-ES.

**Actividad 1.** Untar una tostada	**Ejecución correcta:** Untar la cantidad correcta de mantequilla de manera cuidadosa y uniforme, a un ritmo normal, sin ensuciar y sin provocar situaciones peligrosas con el cuchillo	
*Item 1. Activity: Buttering a sandwich*	*Correct performance: The right amount of butter is neatly and evenly spread, at a normal pace, without making a mess and without dangerous situations involving the knife*	
**Frecuencia** *Mi hijo/a hace esta actividad* 1. Frecuentemente 2. A veces 3. Rara vez 4. Todavía no/nunca	**Calidad** *Mi hijo/a puede hacer esta actividad…* 1. Bien 2. A veces bien y a veces no tan bien 3. No muy bien (o mal) la mayor parte de las veces	**Adquisición** *A mi hijo/a…* Le está llevando o le ha llevado más tiempo aprender a hacer esta actividad que a sus compañeros de su misma edad
*Participation* *My child does this…* *1. Regularly* *2. Sometimes* *3. Seldom* *4. Not yet/never*	*Performance* *My child can do this…* *1. Well* *2. Sometimes well and sometimes not as well* *3. Not very well (or badly) most of the time*	*Learning* *My child…* *Is taking or has taken longer to learn this skill than his/her age peers*
